# *In Vitro* antiproliferative potential of essential oil extract from *Carica papaya* L. seeds against cervical cancer

**DOI:** 10.3389/fphar.2025.1676006

**Published:** 2025-10-02

**Authors:** Doaa Sayed Nady, Sally A. Abdel-Halim, Mohamed-Elamir F. Hegazy, Salwa Aljohani, Aljazi Abdullah AlRashidi, Mohamed A. El-Desouky, Hoda A. Ahmed, Mariusz Jaremko, Abdul-Hamid Emwas, Demiana H. Hanna

**Affiliations:** ^1^ Department of Chemistry, Faculty of Science, Cairo University, Giza, Egypt; ^2^ Chemistry of Medicinal Plants, Department, Pharmaceutical and Drug Industries Research Institute, National Research Centre, Giza, Egypt; ^3^ Department of Pharmaceutical Biology, Institute of Pharmaceutical and Biomedical Sciences, Johannes Gutenberg University, Mainz, Germany; ^4^ Department of Chemistry, College of Science, Taibah University, Madinah, Saudi Arabia; ^5^ Chemistry Department, Faculty of Science, University of Ha'il, Ha'il, Saudi Arabia; ^6^ Department of Chemistry, College of Science in Yanbu, Taibah University, Madinah, Saudi Arabia; ^7^ Division of Biological and Environmental Sciences and Engineering (BESE), Smart-Health Initiative (SHI) and Red Sea Research Center (RSRC), King Abdullah University of Science and Technology (KAUST), Thuwal, Saudi Arabia; ^8^ King Abdullah University of Science and Technology, Core Labs, Thuwal, Saudi Arabia

**Keywords:** *Carica papaya*, essential oil, HeLa cancer cell line, apoptosis, migration, molecular docking

## Abstract

**Introduction:**

Cervical cancer, the third most common cancer worldwide, is primarily caused by human papillomavirus (HPV) infection. This study aimed to develop natural extracts from the seeds of *Carica papaya* L. using the hydrodistillation method to evaluate their anticancer effects against HeLa cells.

**Methods:**

The phytochemical composition of *Carica papaya* seeds’ essential oil was identified using gas chromatography mass spectrometry, and their cytotoxicity on proliferation, apoptosis, cell cycle phases, migration, and colony formation of HeLa cancer cell lines was examined. Moreover, the effects of essential oil on the protein expression levels related to apoptosis, cell cycle regulation, and migration in the treated HeLa cells were examined.

**Result and discussion:**

The essential oil’s phytochemical composition was analyzed using gas chromatography-mass spectrometry (GC-MS), revealing benzyl isothiocyanate as the dominant compound (99.49%). Results demonstrated that the essential oil had a high cytotoxic effect, with an IC_50_ value of 16.78 µg/mL in the MTT assay. Apoptosis analysis indicated a significant increase in early and late apoptotic HeLa cells (23.45%). Flow cytometry revealed a G2/M phase arrest, which impeded cell division. The oil also exhibited a stronger inhibition of cancer cell migration (38.7%) than methotrexate (45.9%). Additionally, the clonogenic assay revealed a drastic reduction in colony formation (0.004% surviving fraction, 0.25% plating efficiency). ELISA results showed a profound effect on apoptosis-related proteins, reducing BCL-2, MMP-2, and CDK1/cyclin B1 expression, supported by molecular docking studies comparing its efficacy to methotrexate (−5.52, −6.29, and −5.75 vs −5.49, −5.44, and 5.18 kcal mol^−^, respectively). The findings suggest that *Carica papaya* seed essential oil may serve as a potential anticancer treatment for cervical cancer; however, further *in vivo* studies are required for validation in animal models.

## 1 Introduction

Cancer remains one of the most significant challenges to public health worldwide, with a profound impact on the healthcare system, individuals, and families (Nady et al., 2023; Chikkanna et al., 2024). Cancer is a group of diseases characterized by the uncontrolled growth and the spread of abnormal cells (Haber et al., 2023). Until now, there has been no absolute care for various types of cancers (Kong et al., 2021; Nady et al., 2024b). Cervical cancer is the third most common type of cancer all over the world, which is mainly caused by human papillomavirus infection (Praveena et al., 2017; Khan, 2021). Worldwide, it is the second most common type of cancer in women after breast cancer (Sari and Handayani, 2020), while in India, it is the leading type of cancer (Khan, 2021). Radiotherapy, surgery, hormone therapy, and chemotherapy are conventional cancer treatment methods (Haber et al., 2023; Jiao et al., 2023; Alzahrani et al., 2024), which are associated with several problems, including high costs (Alzahrani et al., 2024), increasing the multiple resistance (MDR) to drugs, dose-limiting systemic toxicity (Chandrasekaran et al., 2016), and the high cytotoxic effect on normal cells (Alotaibi et al., 2017). The several negative side effects associated with the conventional method have motivated researchers to search for new strategies for cancer treatment. In this context extensive research was focused on utilizing the plant and its extracts for cancer treatment (Varadarajan et al., 2022). Approximately one-third of Food and Drug Administration-approved drugs over the past two decades have been derived from natural products or their derivatives (Zhang et al., 2022).

At present, about 80% of individuals, mainly from developing countries, use traditional medicine derived from medicinal plants (Martin Paul et al., 2019; Abd El-Razek et al., 2023). In cancer treatment, natural compounds make up the majority of approved antitumor medications. Medicinal plants have been especially crucial in discovering and developing new agents for cancer prevention and therapy (Hegazy et al., 2021). Medicinal plants or extracted natural products have shown major advantages over synthetic ones, such as easy availability (Singh et al., 2020), being a cheaper alternative source (Lydia et al., 2016; Abdel-Halim et al., 2021), being more effective (Doughari and Manzara, 2007), having fewer adverse reactions, and having lower toxicity (Zhang et al., 2022), and finally, they offer a rich and renewable reservoir of bioactive compounds, including alkaloids, terpenoids, flavonoids, polyphenols, saponins, and glucosinolates that are essential for the treatment of many diseases (Saha et al., 2023; Hegazy et al., 2016; Varadarajan et al., 2022). One such medicinal plant is *Carica papaya* L.

*C. papaya* belongs to the Caricaceae family, commonly called pawpaw, papaya or papaw (Nirosha and Mangalanayaki, 2013; Doughari and Manzara, 2007; Balavijayalakshmi and Ramalakshmi, 2017). It is native to the tropical and semi-tropical regions of South America and Africa, respectively, but has now spread worldwide (Egbuonu et al., 2016; Abd-elkawy et al., 2019). *C. papaya* is an evergreen-herbaceous plant that looks like a tree but is semi-woody (Abd-elkawy et al., 2019), is marked with lead scars and rarely branches (Egbuonu et al., 2016). The pulp represents around 60% of the weight of *C. papaya* fruit, which is widely used in food products such as jams, juice, ice cream, jelly, and pepin production (Haber et al., 2023). The peel represents 12% of the weight, which is considered agricultural waste, and seeds represent about 20% of the fresh fruit weight, which is also considered agricultural waste (Gaikwad et al., 2023). But nowadays, *C. papaya* seeds are used for many purposes, as an alternative to black pepper, and are characterized by strong antibacterial, antioxidant, anticancer, anthelmintic, liver protection, nephroprotective, and typhoid treatment properties, so the seeds have more potent medicinal effects compared to other *C. papaya* parts (Memudu and Oluwole, 2021). Because of the high medicinal and nutritional values of *C. papaya,* a huge amount is consumed worldwide, which leads to the generation of a large amount of peels and seeds as waste, and these could be considered a significant cause of pollution if not utilized in another aspect (Amran et al., 2021; Phang et al., 2021).

The *C. papaya* is highly cultivated in India, and there are many studies using different parts of *C. papaya* against different cancer cell lines. India is one of the main origins of cultivated papaya around the world, and there are many studies of using Indian papaya extract against different cell lines, first using latex against human breast cancer (MCF-7), which shows a good anticancer response in a dose-dependent manner with IC_50_ equal to 19.88 µg/mL (Chandrasekaran et al., 2016). Using leaf extract against hepatocellular carcinoma (HepG-2) and breast carcinoma (MCF-7, A549, and MDA-MB-231) cell lines (Devanesan et al., 2021; Alzahrani et al., 2024). Also, using seed extracts against oral squamous carcinoma (SCC-25) and the HepG-2 cancer cell line (Anilkumar and Bhanu, 2022), which confirmed the anticancer effect of the seed extract, which exhibited G2/M phase arrest (Varadarajan et al., 2022), along with the potential to induce apoptotic change by downregulation of Bcl-2 protein and upregulation of P53 and Caspase-3 genes (Anilkumar and Bhanu, 2022). Also, pulp and peel extract against colon cancer (Praveena et al., 2017; Vundela et al., 2022), cervical cancer (Praveena et al., 2017), and breast cancer (John et al., 2021; Vundela et al., 2022). Furthermore, the seed extract from *C. papaya* cultivated in Taiwan significantly decreases the cell viability and migration of colorectal cancer cell lines (Chang et al., 2020; Lin et al., 2021). The seed extract from two types of *C. papaya* (California and Bangkok) was able to inhibit the growth of breast cancer cells without causing any toxic effects on normal cells (Alfarabi et al., 2022). Finally, *C. papaya* leaf extract cultivated in Egypt showed a moderate effect against hepatocellular carcinoma (HepG-2) and colon cancer (HCT-116) (Abdel-Halim et al., 2020). While the aqueous seed extract exhibited the anticancer effect against the human colorectal adenocarcinoma cell line (Caco-2) with an IC_50_ equal to 9.73 µg/mL, it regulated the expression of Caspase-3 and P53 genes, triggering cell cycle arrest and apoptosis (Mahrous and Noseer, 2023).

**FIGURE 1:**
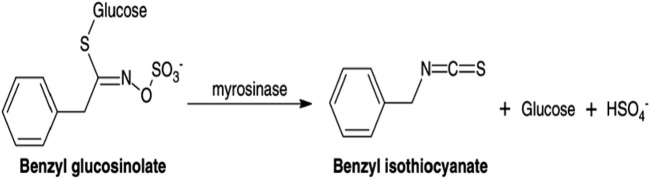
Schematic representation of the enzymatic conversion of benzyl glucosinolate into benzyl isothiocyanate (BITC) catalyzed by myrosinase.

According to previous studies, the fixed oil of *C. papaya* seeds was found to be in a range of 13.9%–30.7%, with a color ranging from pale to dark yellow, and is flavorless and odorless. The most abundant fatty acids are oleic, palmitic, stearic, and linoleic acids (Roberta Malacrida et al., 2011; Yanty et al., 2014), while the essential oil “volatile aromatic” from *C. papaya* seeds has attracted the attention of the scientific community (Komal and Arya, 2013; Yusuf, 2018). Essential oil of *C. papaya* seeds was found to have an amount of benzyl isothiocyanate (BITC) (Nady et al., 2024a), which is mainly formed from benzyl glucosinolate in the presence of myrosinase enzyme, as shown in Figure 1 (Lee et al., 2011).

Recent studies have highlighted the anticancer properties of benzyl isothiocyanate through multiple mechanisms. First, by inducing apoptosis, that is, by promoting programmed cell death by activating Caspase-3 and modulating pro-apoptotic proteins such as anti-apoptotic protein, as Bax (Rahaman et al., 2025; Sinha et al., 2025). By cell cycle arrest, that is, by disrupting the cell cycle progression, especially at the G2/M phase (Sinha et al., 2025). BITC tends to increase the intracellular reactive oxygen species (ROS), which leads to mitochondrial dysfunction and oxidative stress in cancer cells. Also, it influences oncogenic pathways, which are involved in stress response and cell survival (Rahaman et al., 2025)^.^ Finally, BITC has antimetastatic activity, which is by suppressing migration and invasion by downregulating epithelial-mesenchymal transition markers (EMT) and metalloproteinase markers (MMPs) (Sinha et al., 2025).

Although the anticancer potential of *Carica papaya* seed extract has been explored in various studies, the specific impact of the essential oil extracted from *C. papaya* cultivated in Egypt on human cervical cancer cells remains uninvestigated. Therefore, this study aims to characterize the chemical composition of Egyptian *C. papaya* seed essential oil using GC-MS analysis to evaluate its cytotoxic activity against HeLa cervical cancer cells via MTT assay, including the determination of the IC_50_ value. To further elucidate its anticancer effects, the study investigates the essential oil’s effect on apoptosis induction, cell cycle arrest, migration behavior, and clonogenic capacity using different methods such as Annexin V/PI analysis, flow cytometry, wound healing assay, and clonogenic assay, respectively. Additionally, the expression levels of key proteins involved in apoptosis, metastasis, and cell cycle regulation are quantified through ELISA, and their molecular interactions are explored via *in silico* docking analysis. The safety of the essential oil is also examined using the hemolysis assay.

## 2 Materials and methods

### 2.1 Chemicals and reagents

All medium components, chemicals, buffers, solvents, and reagents were purchased from Sigma Aldrich (United States).

### 2.2 Collection and preparation of plant samples

Fresh ripening fruits of *C. papaya* Linn. (family Caricaceae) were obtained from Gardens Resort at Kilometer 63 on the AL-Azizyah Alexandria desert road in July 2022. They were identified and certified by Dr. Kamal Zayed, professor of Ecology at Cairo University, with voucher specimens CAIRC [voucher No. 3237]. The fruits were washed with tap water, followed by distilled water. The fruits were then halved lengthwise, peeled using a kitchen knife, and the seeds were removed. The seeds underwent triple washing with distilled water to eliminate any impurities and dust (Anilkumar and Bhanu, 2022). The seeds were dried at room temperature in shade for 5 days to preserve the seeds’ physicochemical properties (Subbaiah Munagapati et al., 2022). Then dried for 48 h at a temperature of less than 60 °C in a convection oven until constant weight (Yanty et al., 2014; Aina et al., 2020). After that, they were pulverized into fine particles with a size of about 0.85 mm using the electric mill. Following that, the pulverized seeds were stored at (2 °C–3 °C) in a closed container until further use (Sultana et al., 2020). Finally, the percentage of the moisture content and yield were determined using the following Equations 1, 2, respectively (Adesola et al., 2019; Egbuonu et al., 2016):

$$Moisture\;content\;\left(\%\right)=\frac{\text{Weight}\;\text{of}\;\text{wet}\;\text{papaya}\;\text{seeds}-\text{Weight}\;\text{of}\;\text{dry}\;\text{papaya}\;\text{seeds}}{\text{Weight}\;\text{of}\;\text{wet}\;\text{papaya}\;\text{seeds}\;}X\;100$$ (1)

$$Yield\;\left(\%\right)=\frac{Weight\;of\;dry\;seeds\;sample}{\text{Weight}\;\text{of}\;\text{wet}\;\text{seeds}\;\text{sample}}X\;100$$ (2)

### 2.3 Extraction of essential oil from *Carica papaya* seeds

The extraction of EO from *C. papaya* seeds was done utilizing a Clevenger-type apparatus for 3 h, using the previous protocol (Spyridopoulou et al., 2019; Saleh et al., 2020; Mohamed et al., 2022). A batch of 100 g of seeds was mixed with water (200 mL) at 70 °C, then the formed oily layer was extracted using diethyl ether. The collected essential oils were dried over anhydrous sodium sulfate (0.5 g) and then stored at −3 °C in a sealed, airtight glass vial until the next analysis. The weight and volume of the extracted essential oil were detected using the electric balance and pipette, respectively. The yield percentage of extracted oil was determined using the following Equation 3 (Toyin Oshin et al., 2021; Sultana et al., 2020):

$$Oil\;yield\left(\%\right)=\frac{\text{Mass}\;\text{of}\;\text{the}\;\text{extracted}\;\text{oil}}{\text{Mass}\;\text{of}\;\text{the}\;\text{intial}\;\text{seeds}\;\text{sample}\;}X\;100$$ (3)

### 2.4 GC-MS analysis

The extracted essential oils were analyzed using gas chromatography-mass spectrometry (GC-MS) gas chromatography-mass spectrometry. This apparatus consists of a TRACE GC Ultra Gas Chromatograph connected with a thermos mass spectrometer detector. The GC-MS system was outfitted with a TR-5 Ms column, and helium was used as a gas carrier. The program temperature was 60 °C for 1 min, rising by 4 °C/min to 240 °C and fixed for 1 min. The temperature of the detector and injector was kept at 210 °C. The sample was diluted by n-hexane (1:10 v/v), and 1 µL of the diluted mixture was injected (Reda et al., 2017; 2021).

#### 2.4.1 Identification of the essential oil constituents

**FIGURE 2:**
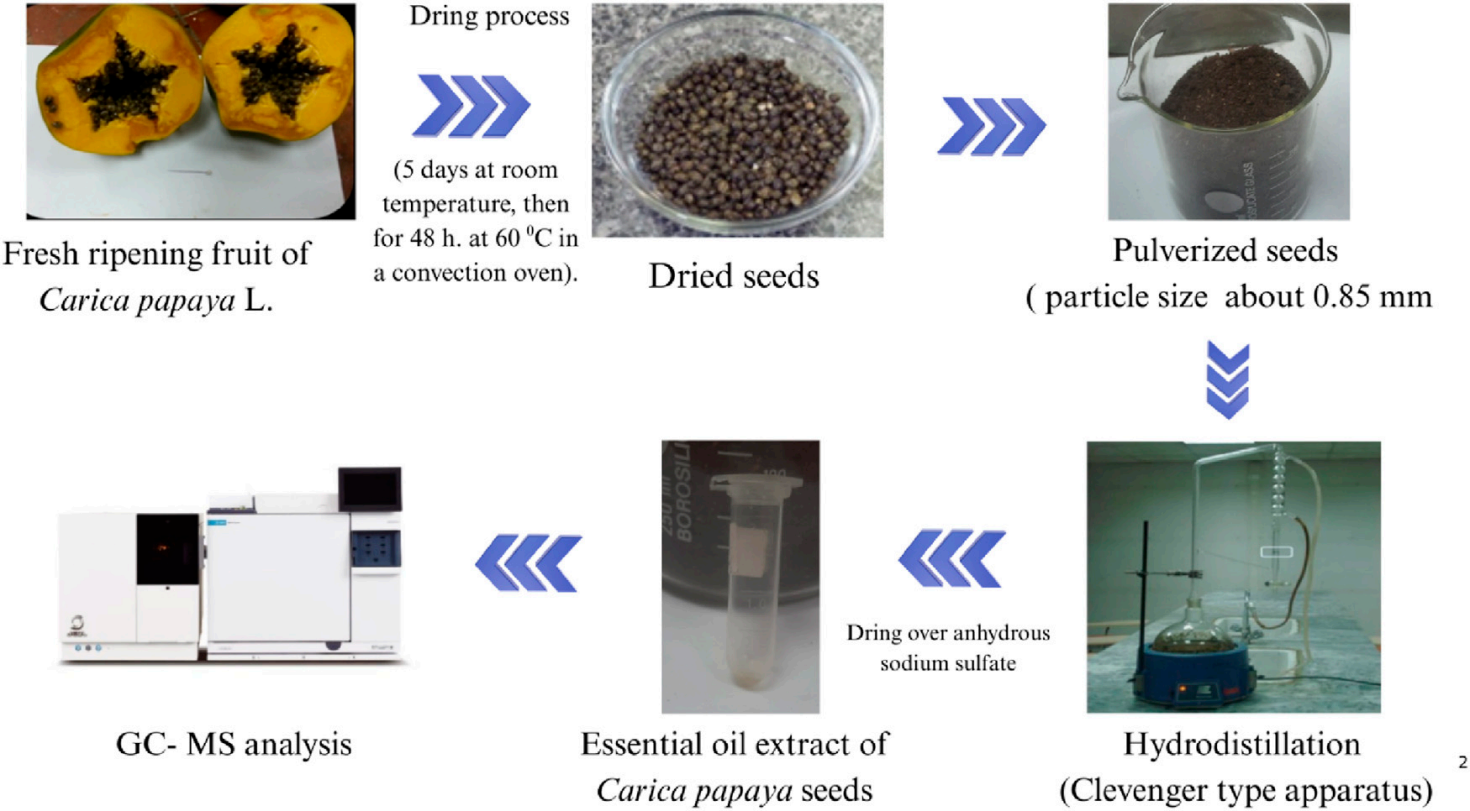
Workflow diagram of *Carica papaya* seed processing and essential oil extraction using the hydrodistillation method.

The chemical constituents of the extracted essential oils were characterized using Automated Mass Spectral Deconvolution and Identification (AMDIS) software and identified by following the Wiley spectral library collection, matching mass spectrum, and NIST library database with authentic standards, as well as their retention indices relative to n-alkanes (C_8_-C_22_) (Reda et al., 2017; Saleh et al., 2020). The mass spectra with those appraisals or published mass spectra with authentic standards are available in the authors’ laboratory (Adams, 2005). The schematic diagram for sample preparation and the extraction process is presented in Figure 2.

### 2.5 Anticancer properties

#### 2.5.1 Preparation and maintenance of cell lines

The Hep-2 cell line (Human Laryngeal Carcinoma) (CCL-23) and the HeLa cell line (Human Cervical Carcinoma) (CCL-2) were purchased from the VACSERA tissue culture unit in Egypt. The cell lines were grown in (DMEM) Dulbecco’s Modified Eagle’s Medium, augmented with (FBS) fetal bovine serum (10%) and supplemented with two antibiotics (penicillin (100 mg/mL) and streptomycin (100 mg/mL)). Then preserved in a humidified atmosphere with 5% CO_2_ at 37 °C. Finally, the cell lines were trypsinized utilizing trypsin EDTA, then subcultured in the culture flasks to keep them and used in the following experiments.

#### 2.5.2 MTT-based cytotoxicity assay

Cell cytotoxicity (cell viability) was detected by [3-(4,5-dimethylthiozol-2-yL)-2,5-diphenyl tetrazolium bromide] (MTT) assay (Moussa et al., 2025b; 2025a; Mustafa et al., 2025). The MTT assay is a colorimetric method used to determine the metabolically viable percentage of Hep-2 and HeLa cancer cells after treatment with the extracted essential oil from *C. papaya* seeds, as previously described (Hanna et al., 2023). In a nutshell, the 96-well tissue culture plate was incubated with 100 µL of cells (1 × 10^5^) per well and incubated for 24 h at 37 °C that is, mainly to develop a complete monolayer sheet. Then, the cells were treated with different concentrations (7.81, 15.62, 31.25, 62.5, 125, 250, 500, and 1,000 µg/mL) of the extracted essential oil at 37 °C for an overnight incubation period. Subsequently, checking for any visual toxicity signs, like cell rounding, granulation, shrinkage, and complete or partial monolayer distribution in both the treated and control cells. After that, the 20 µL of MTT solution (5 mg/mL in PBS) was used to treat untreated cells (control) and the essential oil-treated cells for 4 h at an incubation temperature of 37 °C, that is to allow the metabolism of MTT into formazan which is an insoluble dark purple products that is directly proportional to the viable cells’ numbers. The intracellular reduction of MTT to form the formazan depends mainly on the mitochondrial dehydrogenase enzyme. Then, 200 µL of DMSO (dimethyl sulfoxide) was added to each well with shaking for 5 min at 150 rpm; that is to extract the MTT metabolic product (formazan crystal). Finally, the optical density of the formazan crystal was recorded at 560 nm using a microplate reader (Bio-Rad, United States). The effect of the concentration of the extracted essential oil on the proliferation of HeLa and Hep-2 cancer cells was represented as the percentage of cytotoxicity using the following Equations 4, 5 (Praveena et al., 2017; Anilkumar and Bhanu, 2022; El-Atawy et al., 2024):

$$Cell\;viability\;\left(\%\right)=\frac{\text{Average}\;\text{of}\;\text{the}\;\text{absorbance}\;\text{of}\;\text{the}\;\text{treated}\;\text{cells}\;}{\text{Average}\;\text{of}\;\text{the}\;\text{absorbance}\;\text{of}\;\text{the}\;\text{untreated}\;\text{cells}}X\;100$$ (4)

$$Cytotoxicity\;\left(\%\right)=100-Viability\left(\%\right)$$ (5)

The 50% inhibitory concentration (IC_50_) of the *C. papaya* seeds’ extracted essential oil was determined for 4 h as an incubation period at 37 °C. Where the IC_50_ concentration represents the concentration at which 50% of the cells are still alive.

#### 2.5.3 Annexin V\PI analysis for apoptosis assay

The Annexin V/PI assay can be used to discriminate between necrotic and apoptotic cells using both FITC Annexin V-fluorescein isothiocyanate and (PI) propidium iodide stains, depending on the ability of FITC to bind to the phosphatidyl serine (PS), which allows it to transfer to the outer cell membrane from the inner plasma membrane after the early apoptotic process. Also, the permeabilized membrane of the necrotic cells was identified by using PI. The percentage of apoptotic/necrotic cells in the treated HeLa cells that were previously treated for 24 h with an IC_50_ concentration of the extracted essential oil from *C. papaya* seeds and untreated HeLa cells was detected by an Annexin V-FITC kit (BD Bioscience) as previously reported (Ahmed et al., 2024; Mustafa et al., 2025). In a nutshell, in a 6-well culture plate, HeLa cancer cells were harvested and incubated overnight; that is, it allowed the cells to adhere and proliferate. Then, the HeLa cells were treated with the extracted essential oil for 24 h. Then, the treated and untreated HeLa cells were trypsinized, centrifuged, and then washed with cold phosphate buffer saline twice. After that, the washed cells were reacted with the Annexin V binding buffer, and then into a culture tube, 100 µL of this cell solution was transferred for 15 min in the dark, allowing the Annexin V-FITC to incubate with stained cells and identify the presence of necrotic cells and also early and late apoptotic cells by using a BD Biosciences, BD FACS Calibur^Tm^ flow cytometer.

#### 2.5.4 Examining the different phases of the cell cycle

Cell cycle analysis was examined to identify the cell division phases. The percentage of treated cells with the extracted essential oil and untreated cells through different stages of the cell cycle was evaluated using Abcan, a propidium iodide cytometry kit (catalog ab139418), and a flow cytometry kit, as mentioned earlier (Hanna et al., 2023). Briefly, in the 6-well culture plate, the HeLa cancer cells were harvested, and then the plate was left for 24 h, where the adhesion of cells occurred. Then the cells were incubated with the extracted essential oil for 48 h. After that, the cells were collected, trypsinized with trypsin EDTA, harvested using centrifugation at 1,500 rpm for 5 min, and finally fixed with 70% cold ethanol overnight. After the fixation process, the cells were centrifuged, washed with PBS twice, treated with RNase (100 mM/ml), added to a fluorescent propidium iodide solution, and left in the dark at 37 °C for 30 min, that is, for staining of the treated and untreated cells. In the last step, flow cytometry was used to measure the percentage of existing cells in each phase of the cell cycle (sub-G_1_, G_0_/G_1_, S, and G_2_/M phases) to quantify the DNA content.

#### 2.5.5 Cell migration (the wound healing assay)

Malignant tumors proliferate uncontrollably and can spread throughout the whole body by migrating through the circulatory system. So, a wound healing assay was used to detect how the extracted essential oil can affect the ability of cervical cancer cells (HeLa) to migrate (Hanna et al., 2023). In brief, cells were incubated in a 6-well plate at 37 °C until they confluenced as a monolayer (90%). Then an artificial straight scratch was made by using a pipette tip, simulating a wound at 0 h. The formed scratch was imaged by using a light microscope and then washed with phosphate buffer saline three times. The scratched cells were seeded in fresh media containing the essential oil extracted from *C. papaya* seeds; untreated cells (negative control) were seeded in media containing sterile saline, while the positive control cells were cultured in fresh media containing methotrexate, then they were all incubated for 48 h at an incubation temperature of 37 °C at 5% CO_2_. After the incubation period (48 h), the migration of the negative control, treated cells, and positive control was imaged once more. Finally, the migration rate and the % of wound closure were calculated based on the width of cells scratched at 0 h and 48 h using the following Equations 6, 7, respectively.

$$\text{Rate of cell migration }\left(\text{RM}\right)=\left(w_{i}\;–\;w_{f}\right)\;/\;t$$ (6)

W_i_ = average of the initial width of the wound (µm)

W_f_ = average of final width of the wound (µm)

T = span time of the assay in (hours)

$$\text{Percentage of wound clouser }\left(\%\right)=\left\{\left(A_{t=0}\;–\;A_{t=∆t}\right)\;/\;A_{t=0}\right\}100$$ (7)

A_t=0_ = initial wound area.

A_t=∆t_ = wound area after n hours.

#### 2.5.6 Colonogenic assay

The clonogenic assay is an *in vitro* technique used to assess cell survival, where the capacity of a single cell to proliferate and form a colony is evaluated (Ratnasari et al., 2023). To determine the effects of extracted oil on the clonogenic capacity of HeLa cancer cells, cells were planted in a 6-well plate and incubated for 24 h at an incubation temperature of 37 °C and 5% CO_2_. After the period of incubation, the cells were treated with the extracted essential oil using an IC_50_ concentration, and the culture was then incubated for one to 3 weeks or until a colony was formed. After that, the 6% glutaraldehyde was used to fix the cells, then stained with crystal violet (0.5%) for 30 min. After that, the cells were washed with tap water and then left at room temperature to dry. After the cells were dried, the colonies were observed using a stereomicroscope and a colony counter pen for automatic counting of the colonies. Finally, the plating efficiency (representing the ability to form colonies) and a surviving fraction (SF) were calculated using Equations 8, 9, respectively (El Habbash et al., 2017):

$$Plating\;efficiency\;\left(PE\right)\left(\%\right)=\frac{\text{Number}\;\text{of}\;\text{formed}\;\text{colonies}}{\text{Number}\;\text{of}\;\text{plated}\;\text{cells}}x\;100$$ (8)

$$Surviving\;fraction\;\left(SF\right)\left(\%\right)=\text{PE of treated sample}/\text{PE of control }x\;100$$ (9)

#### 2.5.7 ELISA testing

The expression levels of the proteins involved in apoptosis (Bax, Bcl-2, and Cleaved Caspase-3), cell cycle regulation (Cyclin B1 and CDK1), and metastasis (MMP-9 and MMP-2) in the extracted essential oil-treated and untreated HeLa cells were evaluated and quantified using the ELISA technique. ELISA kits used included Bax (ab199080), Bcl-2 (ab272102), Cleaved Caspase-3 (ab220655), MMP-9 (ab246539), and MMP-2 (ab267813), all provided by Abcam (Cambridge, United Kingdom). These kits employed a sensitive 90-min sandwich ELISA technique with capturing antibodies attached to an affinity tag identified by an anti-tag antibody precoated on the ELISA plates. Treated and untreated HeLa cells were centrifuged, where the pellets were discarded, and the supernatants were used as samples. Equal volumes of the supernatant and an antibody cocktail mixture (containing detector antibodies and capture antibody diluted in Antibody Diluent 5BI) were dispensed into a 96-well ELISA plate and incubated for 1 h at room temperature. After multiple washes with (1X) washing buffer, the substrate solution tetramethylbenzidine (TMB) was added to each well (10 min), and the plate was kept in the dark. Finally, a stop solution was added after that, and the absorbance was measured using an automatic microplate reader at 450 nm. Additionally, the mitosis-regulating protein expression levels were detected using the ELISA Cyclin-Dependent Kinase 1 (CDK1) Kit (Catalog No: MBS458278; MyBioSource, United States) and ELISA Cyclin-B1 Kit (Catalog No: MBS765913; MyBioSource, United States). These kits were based on the sandwich ELISA double-antibody technique, with anti-human CDK1 and anti-human Cyclin-B1 precoated on 96-well plates. Test samples, standards, and biotin-conjugated detection antibodies were added to the wells and then washed using a washing buffer. After that, the wells were treated with HRP (horseradish peroxidase-streptavidin) and TMB substrate, which induced the blue color to turn yellow by adding an acidic stop solution. The yellow color absorbance was measured using an automated microplate reader at 450 nm, which directly correlated with the amount of human CDK1 and Cyclin-B1 in the treated samples, computed using a standard curve.

### 2.6 Analysis of the toxicity of the extracted essential oil using hemolysis assay

The antihemolytic activity of the extracted oil was analyzed using a spectrophotometric approach as previously described (Moussa et al., 2025b). The resulting pellet was washed with phosphate buffer saline (PBS) three times and then immersed in PBS (5% V/V). Then, the erythrocyte solution (400 µL) was added to the extracted essential oil (100 µL) and incubated for 30 min at 37 °C, then centrifuged at RT. At last, the level of hemolysis was measured by determining the absorbance of hemoglobin liberated from cells at 540 nm. The hemolysis percentage was determined using the following Equation 10:

$$Helmolysis\;\left(\%\right)=\frac{A_{\left(test\;sample\right)}-A_{\left(-sample\right)}}{A_{\left(+sample\right)}-A_{\left(-sample\right)}}X\;100$$ (10)

where A_(test sample)_ represents the optical density of the sample containing the extracted essential oil, A_(+sample)_ represents the absorbance of the positive control (containing erythrocytes treated with Triton X-100), and A_(-sample)_ represents the absorbance of the negative control (containing erythrocyte solution in PBS only).

### 2.7 Molecular docking process

The structure of benzyl isothiocyanate, as well as the positive control, methotrexate (MTX), was downloaded as an SDF file from the PubChem database (https://pubchem.ncbi.nlm.nih.gov/). Then, converted SDF files to PDB format by using the free software Avogadro (https://avogadro.cc/). The PDB file for BCL-2 crystal structures (PDB ID: 1K3K) is a key player for pro- or anti-apoptotic activities, the PDB file for MMP-2 crystal structures (PDB ID: 1HOV) is a key player in the tumor ecosystem, and the PDB file for CDK1/cyclin B1 crystal structures (PDB ID: 4YC3) is a key player for arrest in G2/M. They were downloaded from the Protein Data Bank (http://www.rcsb.org/pdb/). These targets were selected based on their biological relevance: BCL-2 as a regulator of apoptosis, MMP-2 as a modulator of the tumor microenvironment, and CDK1/Cyclin B1 as a checkpoint controller of the G2/M cell cycle arrest, supported by prior literature (Petros et al., 2001; Quick et al., 2014; Brown et al., 2015; Hobani et al., 2017). As co-crystallized ligands were not available for 1K3K and 1HOV structures, blind docking was initially performed to identify potential binding regions. The convergence of benzyl isothiocyanate poses across multiple runs informed the prediction of the active site, which was Docking box dimensions were defined around this predicted site to ensure accurate redocking and pose evaluation. The box dimensions for 1K3K are 230.7 mm, 32.8 mm, and 116.9 mm for X, Y, and Z, respectively, while the box dimensions for 1HOV are 17.1 mm, 26.6 mm, and 19.7 mm for X, Y, and Z, respectively. The CDK1–Cyclin B1 complex (PDB ID: 4YC3) contains a co-crystallized ligand, enabling structural validation of the binding pocket. The active site was further predicted using Proteins Plus (https://proteins.plus), integrating geometric and chemical criteria to refine docking box dimensions and ensure accurate ligand placement. The box dimensions are 22.924, 5.297, and 168.970. Validation procedures included pose clustering analysis based on RMSD similarity and binding energy ranking. These metrics were used to assess conformational consistency and identify the most stable ligand orientations.

Then, converted SDF files to PDB format by using the free software Avogadro (https://avogadro.cc/). To calculate the standard deviations and mean value of the lowest binding energies, the docking process was performed three times independently. Validation procedures included pose clustering analysis based on RMSD similarity and binding energy ranking to assess conformational consistency and identify the most stable ligand orientations. The representation and graphical analyses were performed using the BIOVIA Discovery Studio Visualizer software. This study was used to prepare molecular docking simulations and protein structure by using AutodockTools (1.5.6rc316) software. Docking was performed using AutoDock4 using the Lamarckian algorithm as described before (Fukaya et al., 2018).

### 2.8 Statistical analysis

Each study was conducted three times, and the results are presented as mean ± standard deviation. Independent T-tests were used for all statistical analyses, performed with SPSS 17.0 software. Statistical significance was defined for p-values of p ≤ 0.05, p ≤ 0.01, and p ≤ 0.001.

## 3 Results and discussion

### 3.1 Physicochemical properties of the extract

The quantity and quality of the extracted essential oil and other phyto-constituents of the natural extract (e.g., *Carica papaya* seed) differ according to the origin, seasonal variation, and ripening level, as well as the extraction technique (Shaheen et al., 2023). In this study, *C. papaya* seeds showed a high percentage of yield (67.36%), where the initial wet seeds weight is 297.1 g and the dry seeds weight is 200.12 g. Also, the moisture content of seeds was calculated to be equal to 32.53%. Besides the extraction process, the moisture content and drying process before the oil extraction from the seeds play a role in the yield of extracted oil (Tan, 2019). The *C. papaya* seeds’ essential oil extracted using the hydrodistillation extraction method was yellow, and the overall yield (based on the weight of dried seeds w/w (100 g)) was equal to 0.12%. In another study, the overall yield of extracted essential oil from *Carica papaya* seeds from China using the hydrodistillation method was 0.2% (w/w, based on the weight of dried seeds (300 g)) (He et al., 2017).

Most of the previous studies of the fixed oil content of *C. papaya* seeds showed that the fixed oil (fats) content of *C. papaya* seeds ranges between 13.9% and 30.7%, and the color ranges from pale to dark yellow and is almost flavorless and odorless (Yanty et al., 2014). Also, they found that the functional and nutritional properties of fixed oil extracted from *C. papaya* seeds are highly similar to olive oil (Tan, 2019). It has a high level of monounsaturated fatty acids (MUFA) and a low level of total tocopherol (Roberta Malacrida et al., 2011), so *C. papaya* seed oil represents a good prospective source of oil (Tan, 2019). Nowadays, the extracted essential oil has attracted a lot of attention due to having many applications in different fields like food, cosmetics, industry, and medicine. That EOs have several biological activities, including antitumor, anti-inflammatory, antiviral, antioxidant, and antibacterial activity (Shakeri et al., 2016; He et al., 2017; Yusuf, 2018).

#### 3.1.1 Chemical composition of essential oil

**FIGURE 3:**
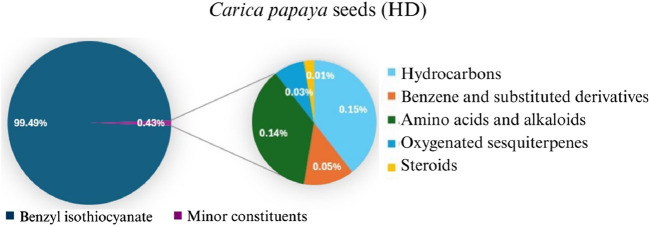
Pie chart illustrates the chemical composition of *Carica papaya* seeds extract obtained via hydrodistillation. Benzyl isothiocyanate constitutes the major component (99.49%), while minor constituents, including hydrocarbons, benzene derivatives, amino acids, oxygenated sesquiterpenes, and steroids, collectively account for 0.43% of the total composition.

**FIGURE 4:**
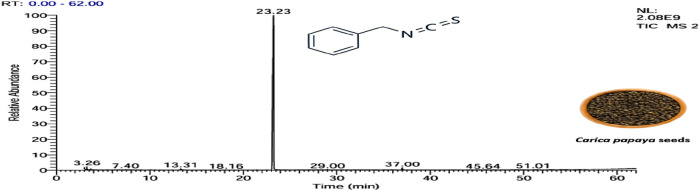
Essential oil extracted from *Carica papaya* seeds, as shown by the GC–MS.

The yield of the extracted essential oil was 0.12% v/w (yellow), which was obtained after the extraction of oil from *C. papaya* seeds using hydrodistillation. Compound identification was achieved based on their mass spectral data (MS) to compare with the NIST library databases, the Wiley spectral library collection, and GNPS. There were an estimated 99.87% of total pointed-out molecules, Figure 3. The chemical components of the extracted essential oils, along with their retention times (RT), experimentally determined Kovats retention indices (KI Exp.), and Kovats retention indices from literature, are summarized in Table 1. The essential oil’s chemical composition closely matches previous studies, predominantly consisting of isothiocyanates (ITCs). Benzyl isothiocyanate (BITC) is the primary isothiocyanate, representing 99.49% of the composition (He et al., 2017), as shown in Figure 4. Isothiocyanates (ITCs), present in cruciferous vegetables like broccoli and cabbage, possess a range of properties, including antibacterial, antifungal, antioxidant, and cytoprotective effects (Ramakrishnan et al., 2024). The rest of the classes of essential oil constituents are present as traces (0.38%). They were identified as hydrocarbons (0.15%), alkaloids (0.02%), amino acids (0.12%), oxygenated sesquiterpenes (0.03%), and steroids (0.01%). Their biological activities are mentioned in Table 1.

**TABLE 1:** Identification of bioactive constituents in the essential oil of *Carica papaya* seeds obtained via hydrodistillation, as determined by GC-MS analysis. The table highlights the major compounds with known biological activity, including their retention times, molecular formulas, and pharmacological activity.

No.	RT (min)	Compound name	MF	MW (g\mol)	Structure	K_Lit_	K_Exp_	Peak area (%)	Base peak (m/z)	Key fragments (m/z)	Biological activity	Reference
Hydrocarbons												
1	3.02	Heptane	C_7_H_16_	100	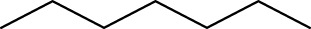	700	695	0.02	43	71, 57, 41		
2	3.26	Methylcyclohexane	C_7_H_14_	98	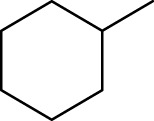	720	212	0.13	83	98, 69, 55, 41	-	(Macleod and Pieris, 1983)
3	3.65	Toluene	C_7_H_8_	92	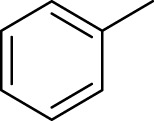	770	763	0.05	91	92, 65, 51, 39	-	Macleod and Pieris (1983)
Total hydrocarbons								0.20				
Alkaloids												
4	13.32	2-phenylacetonitrile	C_8_H_7_N	117	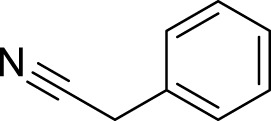	1,148	1,145	0.12	117	116 90, 51	-	(Macleod and Pieris, 1983; MacLeod and Pieris, 1983)^[2][4]^
5	23.23	Benzyl isothiocyanate	C_8_H_7_NS	149	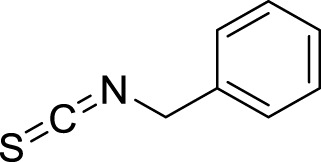	1,353–1,371	1,360	99.49	91	92, 65, 61	Anticarcinogenic, anthelmintic, and antibacterial activity	(Chandra Kala and Ammani, 2017; Dahham et al., 2015)
6	43.47	Pterin-6-carboxylic acid	C_7_H_5_N_5_O_3_	207	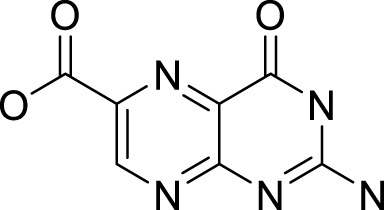	2,453	2,455	0.02	44	207, 163, 149	Anti-Tumor Activity	(Ananthanayagi et al., 2022; Dinh et al., 2021; Li et al., 2021)
Total alkaloids								99.63				
Oxygenated sesquiterpenes												
7	35.31	Caryophyllene oxide	C_15_H_24_O	220	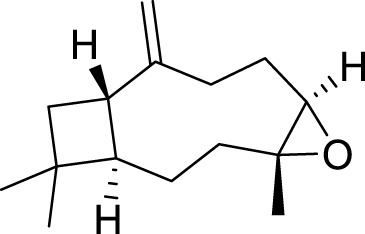	1,583	1,580	0.01	43	220, 121, 79	Antioxidant, anticancer antibacterial, anti-inflammatory and anesthetic activity	(Ulubelen et al., 1994; Kadhim et al., 2016; Almanaa et al., 2022; Youssef et al., 2023)
8	35.53	*tau*-Cadinol	C_15_H_26_O	222	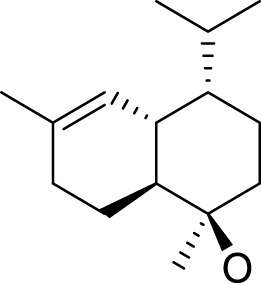	1,640	1,635	0.02	161	204, 189, 134	Antibacterial activity	Abd-ElGawad et al. (2022)
Total Oxygenated Sesquiterpenes								0.03				
Steroids												
9	47.76	Ethyl iso-allocholate	C_26_H_44_O_5_	436	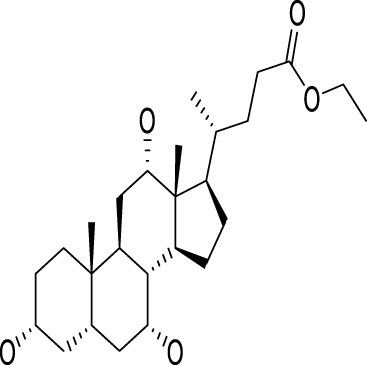	2,478	2,477	0.01	354	400, 280, 200	Antioxidant, antimicrobial, anti-inflammatory, anti-arthritic, and antiasthmatic activity	(Elsharkawy et al., 2021; Taha Mohamed et al., 2021)
Total Steroids								0.01				
Total identified compounds								99.87				
Total unidentified compounds								0.13				

RT, is the retention time, MF, is the molecular formula, Mw is the molecular weight, Ki_lit_ “is the published Kovats Retention indices”, Ki_exp_ “is the Kovats index determined experimentally relative to n-alkanes (C_8_-C_28_)” and indicates no data available.

The identification of benzyl isothiocyanate as a major compound in the extracted essential oil from papaya seeds. Consistent with the research of He et al. (2017), who demonstrated that three components were identified in the extracted essential oils from papaya seeds. The major compound was BITC, representing 99.36% of the total extract, and the other two compounds are benzyl nitrile and benzaldehyde, accounting for 0.31% and 0.33%, respectively (He et al., 2017). Also, this is in agreement with Yi et al. (2022), who reported BITC as a mainly chemical component of the extracted essential oils of *C. papaya* seed from China, with a content accounting for 97.43% (Yi et al., 2022).

BITC is a bioactive compound that has been used in different areas because of its wide applications, which range from vascular relaxation to cancer proliferation inhibition (Toyin Oshin et al., 2021; Barroso et al., 2016). Studies on the effect of BITC as a pure compound or extracted from natural products against different types of cancer such as lung cancer, prostate cancer, leukemia, colon cancer, breast cancer, hepatocellular carcinoma, and pancreatic cancer (Ranjan et al., 2019), reported that BITC has anticancer activities that are mediated by modulation of various signaling pathways, including cell proliferation, cell migration, cell cycle arrest, apoptosis, and invasion (Lee et al., 2011; Yi et al., 2022). However, the application of BITC in food, medicine, industry, and other fields is restricted by its easy degradation, instability, volatility, and poor water solubility (Zheng et al., 2025).

### 3.2 Efficiency of the prepared oil extract on the proliferation of the HeLa cancer cell line

Nowadays, it is vital to replace synthetic anticancer agents with natural ones. Therefore, the current work aims to investigate the essential oil extracted from *C. papaya* seeds, composed mainly of benzyl isothiocyanate (aromatic isothiocyanate), as mentioned above. Benzyl isothiocyanate is considered to be the hallmark of cancer, which is due to its ability to modulate various signaling pathways, including cell cycle arrest, apoptosis, and cell proliferation (Ranjan et al., 2019; Yi et al., 2022).

#### 3.2.1 MTT cytotoxicity assay

**FIGURE 5:**
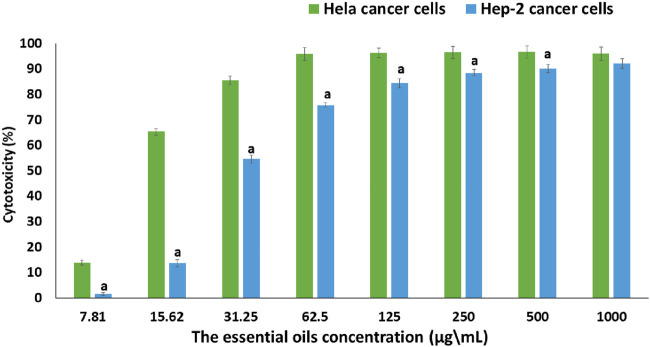
The cytotoxic effect of extracted essential oils at various concentrations (7.81–1,000 µg/mL) on HeLa and Hep-2 cancer cell lines using the MTT test. Data are presented as mean ± SD of three separate analyses. The statistical significance was assessed using an independent T-test to compare the studied groups. Statistically significant (*p* < 0.05) differences are shown by lowercase letters. a: (*p* < 0.05) with respect to HeLa cancer cell lines.

**FIGURE 6:**
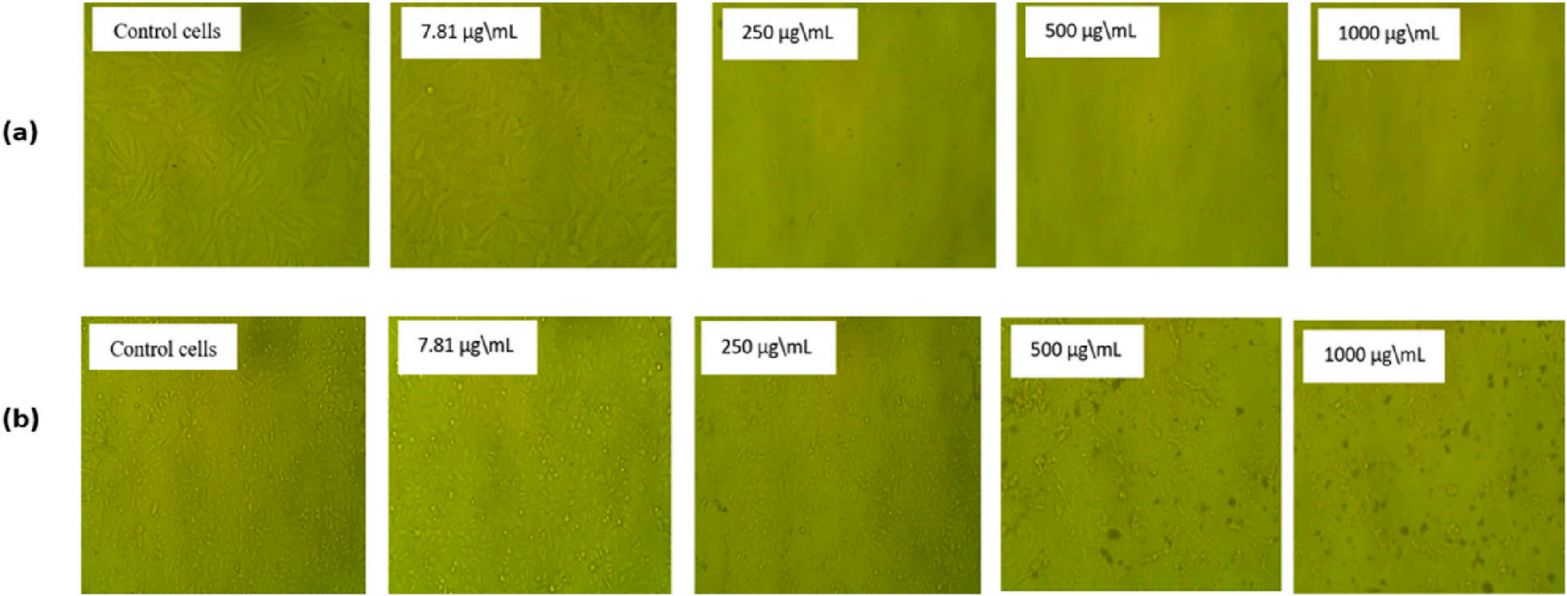
The morphological alterations in HeLa cells **(a)** and Hep-2 cells **(b)** after 24 h treatment with various concentrations of the extracted essential oil compound compared to normal control cells. The control group of both cell lines has not been treated and has a regular form. **(b)** show minimal morphological alterations in Hep-2 cells treated with extracted essential oil, in contrast to **(a)**, which showed substantial shrinkage of cells and detachment, indicating cytotoxic impacts, especially for 1,000 µg/mL, showing a significant number of cell deaths, which means that the extracted essential oil effectively eliminates cervical carcinoma cells in greater quantities.

The MTT assay is used to investigate the *in vitro* cell viability assay, which is important to measure the cellular response to the drug or toxicant and gives information about cell death, survival, and metabolic activity (Chandrasekaran et al., 2016). The present study aimed to examine the antiproliferative activity of the extracted essential oil against two types of human cancer cells and determine the effective concentration (IC_50_) that causes a 50% loss of cell viability. The efficacy of the extracted essential oil on the growth and morphology of Hep-2 and HeLa cancer cells using the MTT assay is represented in Figures 5, 6, respectively.

By comparing with the control untreated cells (Figure 5), the percentage of cytotoxicity was significantly increased with increasing concentration of the extracted essential oil in both cell lines (7.81–1,000 µg/mL), where the extracted essential oil showed a stronger cytotoxicity effect against HeLa cells than Hep-2 cells at each tested concentration. So, at the highest concentration (1,000 µg/mL), the percentage of cytotoxicity on the HeLa and Hep-2 cells was found to be 96.07% ± 0.001% and 92.16% ± 0.002%, respectively. Also, at the lowest concentration (7.81 µg/mL), the percentage of cytotoxicity of the extracted oil on the HeLa and Hep-2 cells was found to be 13.87% ± 0.01% and 0.23% ± 0.01%, respectively. Moreover, the extracted oil had a stronger IC_50_ value (16.78 ± 0.38 µg/mL) against the HeLa cell line than the Hep-2 cell line (37.84 ± 0.64 µg/mL). According to the National Cancer Institute’s guidelines and criteria, if the IC_50_ of the tested sample had an amount less than 20 µg/mL, it was classified as a potent sample for cancer cell treatment.

The morphological change of the cells, such as rounding or shrinking of the cells, granulation, and vacuolization in the cytoplasm of the cell, can be detected on direct microscopic observation and is considered an indicator of cytotoxicity (Praveena et al., 2017). As displayed in Figure 6, in comparison to the untreated cells, which showed a strong adherent cell to the surface and developed on an epithelial monolayer, the extracted essential oil showed higher morphological apoptotic variations in HeLa cells (Figure 6a) in Hep-2 cells (Figure 6b), such as cell shrinkage, phase-dense nucleus, rounded cell, and a decrease in the number of cells by increasing the extract concentration. By comparing with the control untreated cells, the percentage of cell viability was significantly decreased with an increase in concentration of the extracted essential oil (7.81–1,000 µg/mL) in both cell lines. At the highest concentration (1,000 µg/mL), the percentage of cell viability on HeLa and Hep-2 cells was found to be 3.93% ± 0.001% and 7.85% ± 0.002%, respectively. Also, at the lowest concentration (7.81 µg/mL), the percentage of cell viability of the extracted oil on HeLa and Hep-2 cells was found to be 86.13% ± 0.01% and 99.77% ± 0.01%, respectively.

In the past, various studies evaluated the anticancer potential of the different *C. papaya* extracts against different cell lines. First, different studies investigate the cytotoxic effect of different *C. papaya* extracts against different hepatocellular cancer cell lines. Based on a study conducted by Tessy John et al., the *C. papaya* peel extract loaded on silver nanoparticles has a cytotoxic effect against the Hep-2 cell line with an IC_50_ equal to 83.06 µg/mL (John et al., 2021). Also, different studies evaluated the effect of different *C. papaya* extracts, like fruit juice, lycopene, leaves, and seeds, on the Hep-2 cell line, which showed a significant inhibition in a dose-dependent manner (Salla et al., 2016; Abdel-Halim et al., 2020; Devanesan et al., 2021; Anilkumar and Bhanu P A, 2022).

Second, various investigations evaluated the anticancer potential of various papaya extracts against the cervical cancer cell line. Thanigaimalai et al. (2025) investigated the cytotoxic effect of ethanolic extract of *Carica papaya* leaves, roots, and seeds, which inhibited the proliferation of HeLa cell lines up to 19.57%, 25.76%, and 29.21%, respectively, at 100 µg/mL concentration. The IC_50_ value of the ethanolic leaf extract is found to be about 19.57 ± 0.048 µg/mL, indicating a greater cytotoxic effect in cervical cancer cells (Thanigaimalai et al., 2025). In another study, the *in vitro* cytotoxic study of *C. papaya* bark extract on the HeLa cancer cell line showed a dose-dependent cell death with an IC_50_ value of 18 ± 0.4 µg/mL, compared to Doxorubicin with an IC_50_ value of 11 ± 0.5 µg/mL (Altamimi et al., 2025).

#### 3.2.2 Estimation of the effect of oil extract on cell apoptosis

**FIGURE 7:**
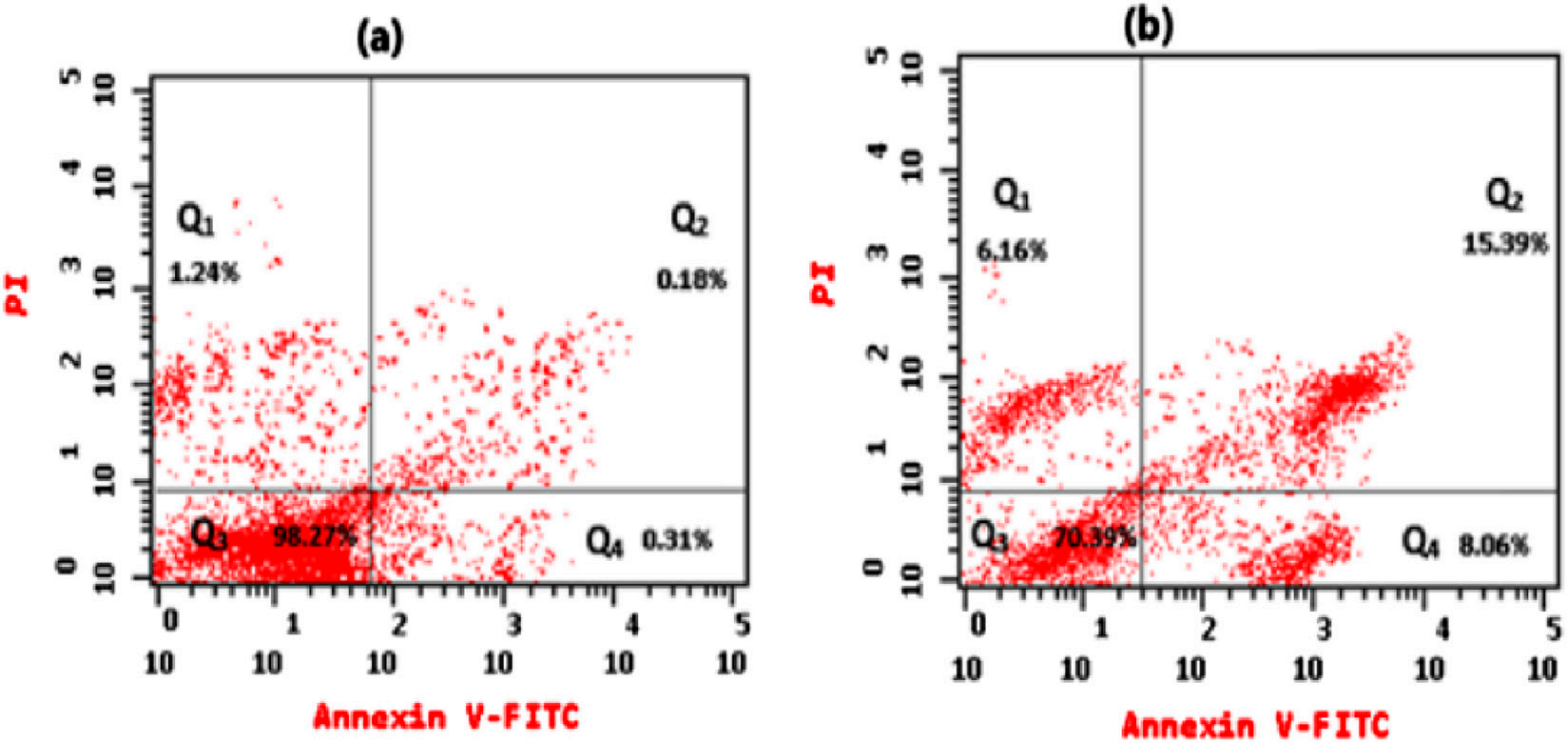
Flow cytometry assessed the apoptotic pattern of HeLa cells, showing Q1 (An^−^, PI^+^) percentage of necrotic cells, Q2 (An^+^, PI^+^) percentage of late apoptotic cells, Q3 (An^−^, PI^−^) percentage of viable cells, and Q4 (An^+^, PI^−^) percentage of early apoptotic cells for treated cells with an IC_50_ dose of essential oil for 24 h **(b)** in comparison to untreated HeLa cells **(a)**.

**FIGURE 8:**
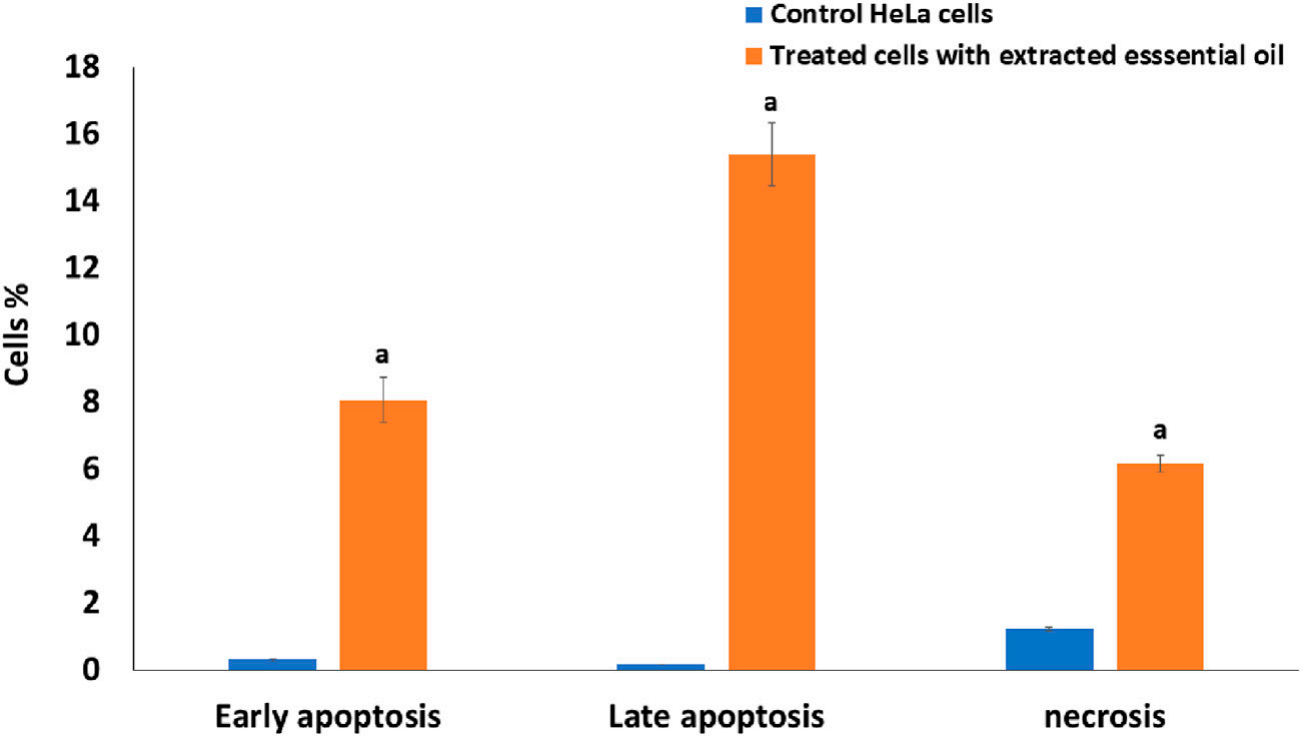
Comparative analysis of early apoptosis, late apoptosis, and necrosis percentage in HeLa cells treated with IC_50_ of the extracted essential oil for 24 h using the Annexin V-FITC/PI staining method versus untreated control cells. The statistical significance was assessed using an independent T-test to compare the studied groups. Statistically significant (*p* < 0.05) differences are shown by lowercase letters. a: (*p* < 0.05) with respect to the control untreated cells.

Cancer cells proliferate excessively, can survive in an overstretched environment, and have a high ability to resist therapies. Whereas conceiving the tumor cells’ apoptosis has appeared as a useful treatment method for cancer (Alzahrani et al., 2024). By using Annexin V-FITC/PI staining, the proportion of apoptosis in the untreated cells (control) and treated cells with extracted oil was investigated. As seen in Figures 7, 8, there was a significant increase in the percentage of necrotic, early apoptotic, and late apoptotic cells in the HeLa cancer cells treated with the extracted essential oil (6.16%, 8.06%, and 15.39%, respectively) when compared to control cells (1.24, 0.31%, and 0.18%, respectively). The results indicated that the extracted essential oil from *C. papaya* seeds could induce the apoptosis pathway in HeLa-treated cells.

The Annexin V/PI flow cytometry assay revealed that the extracted essential oil primarily induced apoptosis, as evidenced by the substantial increase in early apoptotic cells (15.39%) compared with control cells (0.18%). While a necrotic population of ∼8.06% was detected, this is most likely attributable to secondary necrosis, a common outcome of apoptosis *in vitro* when apoptotic bodies are not removed by phagocytosis (Galluzzi et al., 2015; Costigan et al., 2023; Berghe et al., 2014). These findings indicate that programmed cell death via apoptosis represents the predominant mechanism of cytotoxicity induced by the essential oil, while necrosis constitutes only a minor secondary event. Several essential oils have been shown to trigger cell death primarily through apoptotic pathways, with necrotic cell death playing only a minimal role. For instance, Pallines spinosa flower and leaf essential oils (F-PSEO, L-PSEO) induced apoptosis in MCF-7 and MDA-MB-231 breast cancer cells—80%–90% and 30%–50%, respectively —with necrosis remaining below 10% (Berghe et al., 2014; Galluzzi et al., 2015; Costigan et al., 2023).

#### 3.2.3 Cell cycle analysis

Cell cycle regulation is the elementary process for organizing cell proliferation, where cells have three checkpoints: G_1_ (gap 1), S (synthesis of DNA), and G_2_/M (mitosis, gap 2) as control mechanisms to ensure that the switch from one phase to another occurs in an ordered way (Alzahrani et al., 2024). The major characteristic of cancer cells is the disruption of the cell cycle flow (Alzahrani et al., 2024), so using flow cytometry is considered a possible analysis to find the impact of the extracted essential oil on regulating the HeLa cell cycle phases.

**FIGURE 9:**
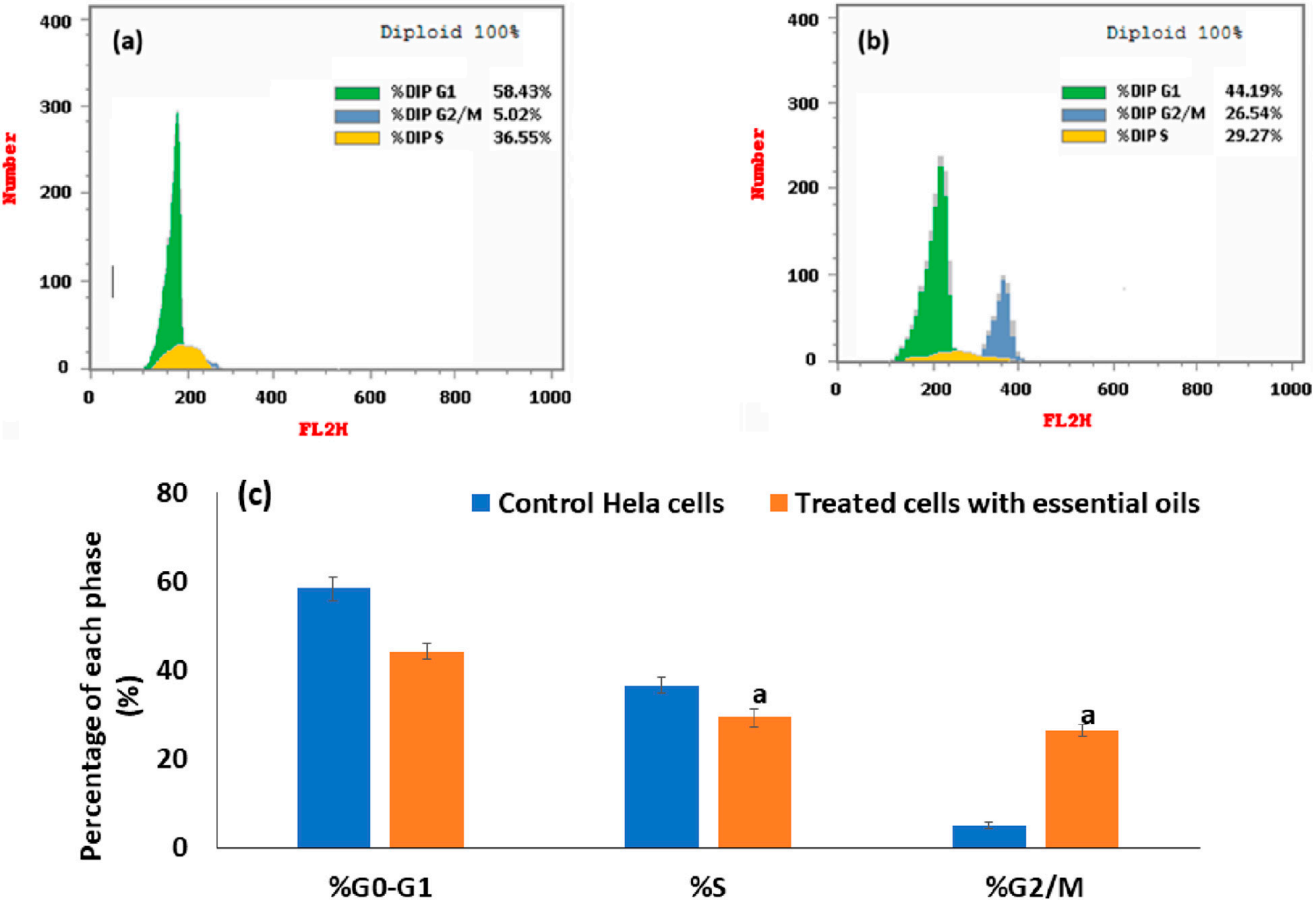
Flow cytometric analysis of the distribution of the cell cycle at different stages in control cells **(a)** and in HeLa cells that had been treated with an IC_50_ dose of *essential oil* for 24 h **(b)**. An illustration of a histogram that displays the percentage of cells in each stage of the cell cycle in untreated and essential oil-treated cells **(c)**. The statistical significance was assessed using an independent T-test to compare the studied groups. Statistically significant (*p* < 0.05) differences are shown by lowercase letters. a: (*p* < 0.05) with respect to the control untreated cells.

As shown in Figure 9, there was a significant increase in the cell percentages in the extracted essential oil-treated HeLa cells (Figure 9b) in the G_2_/M phase (26.54%) when compared with the percentages of cells in the control cells (Figure 9a) (5.02%), P < 0.001. But there was a significant decrease in the treated HeLa cells with extracted essential oil in G_0_/G_1_ and S phases (44.19% and 29.27%, respectively) when compared to control cells (58.43% and 36.55%, respectively), P < 0.001. Also, the percentages of the cells in each phase of the cell cycle in the extracted treated HeLa cells and control cells are represented in a representative histogram as shown in Figure 9c. The results revealed that the extracted essential oil induced the cell cycle arrest (DNA accumulation) at the G_2_/M phase in HeLa cancer cells. In a previous study, Praveeno P. et al demonstrated the effect of hydroethanolic extract of unripe fruit of *C. papaya,* where the extract showed high activity against cervical cancer cell lines, suggesting the inhibition and the interaction of various components participating in cell cycle regulation, and the IC_50_ value was found to be equal to 75.88 µg/mL (Praveena et al., 2017). Also, different extracts of *C. papaya* leaves showed a cytotoxic effect against HeLa cancer cells in a concentration-dependent manner (Bhuiyan et al., 2020; Haber et al., 2023).

#### 3.2.4 Effectiveness of the extracted essential oil in preventing HeLa cells metastasis

Metastasis is a complex process that occurs through several interconnected processes, such as migration, invasion, and adhesion of cancer cells. So, it has been hypothesized that interfering with the motility, adherence, and invasion of the tumor cells to the target organ is one way to inhibit the cancer spread. The ability of cancer cells to migrate is considered the essential process for cancer invasion and metastasis (Spyridopoulou et al., 2019). Therefore, using a wound healing assay, this study investigated whether the extracted essential oil from the *C. papaya* seeds could attenuate the HeLa cervical cancer cell migration rate *in vitro.*

**FIGURE 10:**
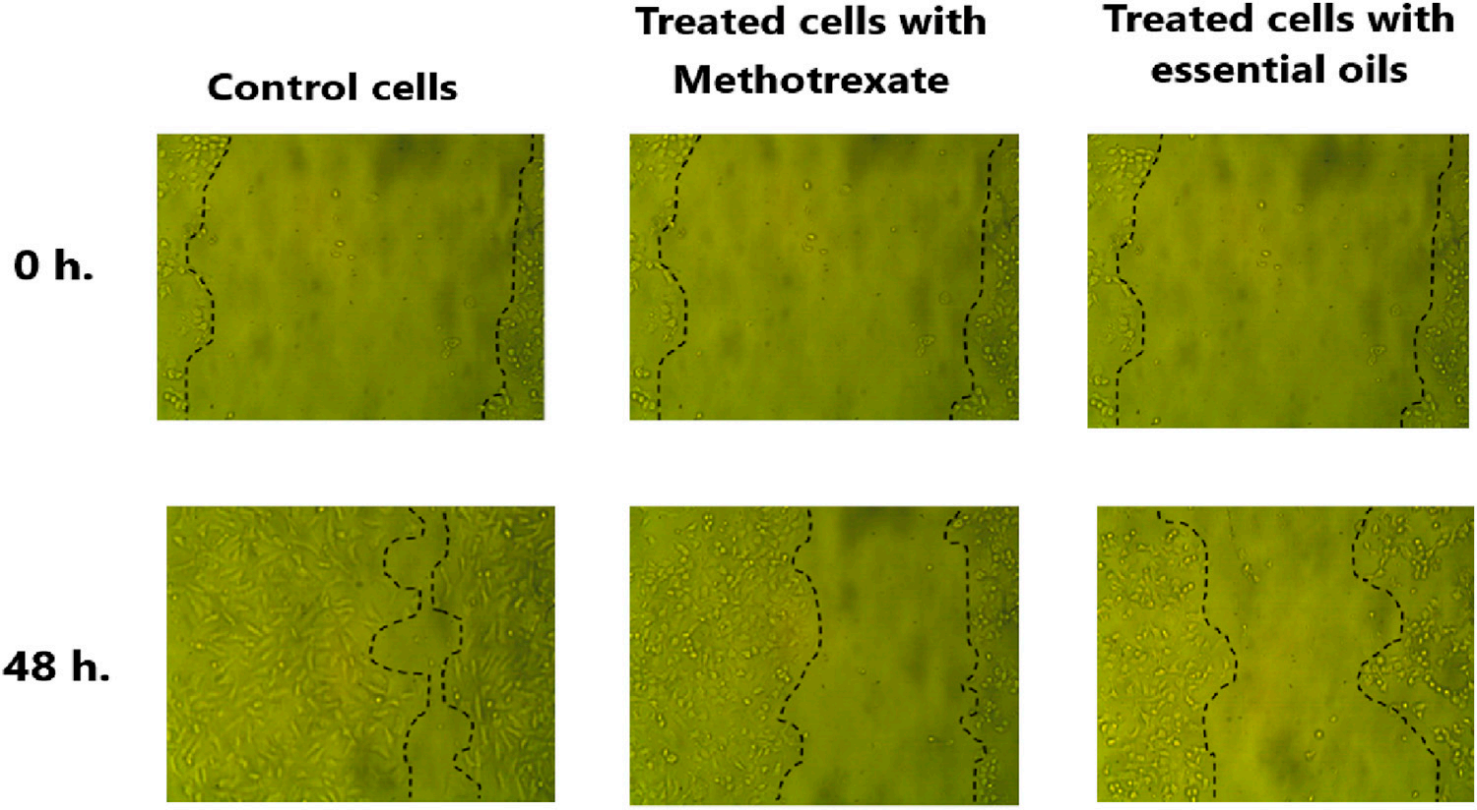
Microscopic examination of HeLa cells’ migration was assessed via a wound healing assay over a 48 h period. After scratching the HeLa cells, the migration of untreated cells (control), treated cells with methotrexate (positive control), and treated cells with extracted essential oil to the wound area was imaged at 0 h and following 48 h. The dashed lines mark the wound boundaries, and the degree of closure reflects the migration response under each condition.

The attained results indicated that the open area (wound) closed earlier in untreated cells (negative control) with a migration rate of 14.1 µm compared to the migration rate in the cells treated with methotrexate, positive control (7.89 µm), and the cells treated with the extracted essential oil (6.66 µm). As shown in Figure 10, the percentage of wound closure was 38.78% and 45.9% in scratched HeLa cells treated with essential oil and methotrexate, respectively compared to the wound closure % in the untreated cells (81.98%).

#### 3.2.5 Clonogenic assay

**FIGURE 11:**
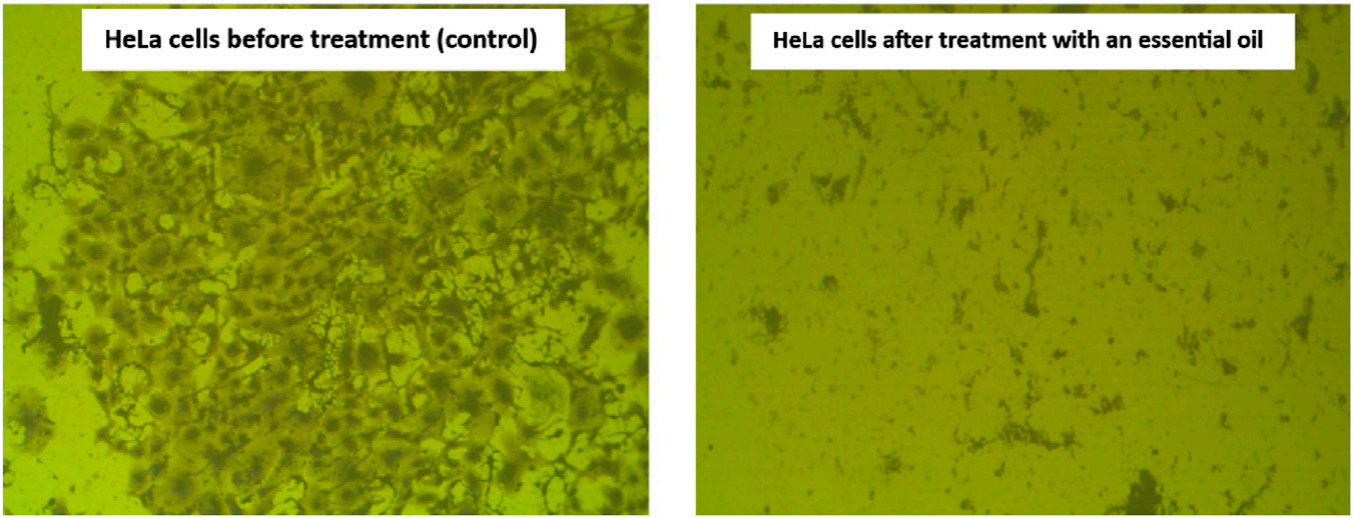
Microscopic examination demonstrating the effect of the extracted essential oil on HeLa cells’ survival. The control cells show a dense colony formation, while the treated cells with extracted oil display a marked reduction in colony density. This reduction reflects the inhibitory effect of the extracted oil on cell proliferation and viability.

The clonogenic assay is an *in vitro* cell survival assay that depends mainly on the ability of a single cell to form a colony. In contrast, it is the only assay that monitors the ability of cancer cells to form a viable colony (Ratnasari et al., 2023). Commonly, the cancer cells have a proclivity to grow in colonies, where the loss of the connection between cancer cells and the adjacent cells leads to the death of the cancer cells. The obtained results reported that the extracted essential oil significantly inhibits the ability of HeLa cells to form colonies with a plating efficiency (PE) of 0.25% ± 0.29% and a surviving fraction (SF) of 0.004% ± 0.29% compared to the plating efficiency (61 ± 1.49) and the surviving fraction (1%) of HeLa cells before treatment. Moreover, using a stereomicroscope, the examination of the plating efficiency and survival of essential oil on the HeLa cells was displayed in Figure 11, showing a significant decrease in density and number of the HeLa cells after treatment with the extracted essential oil compared to the HeLa cells before treatment.

In the clonogenic assay, treatment with the essential oil at the IC_50_ concentration (determined by the MTT assay) resulted in almost complete ablation of colony formation (SF = 0.004%). This outcome reflects loss of reproductive viability, a recognized hallmark of cytotoxicity (Franken et al., 2006), rather than a nonspecific effect of excessive dosing. Supporting this interpretation, Annexin V/PI analysis demonstrated apoptosis as the predominant mode of death (21.55% apoptotic vs 8.06% necrotic), while cell cycle analysis revealed G_2_/M arrest. Furthermore, migration assays indicated suppression of motility, suggesting a cytostatic contribution. These findings align with previous reports that essential oils exert anticancer effects mainly through apoptosis induction with ancillary cytostatic activity (Bayala et al., 2014; Edris and Fairag, 2003). Collectively, the results demonstrate that the essential oil induces an apoptosis-predominant cytotoxic response with additional cytostatic effects, thereby explaining the dramatic reduction in clonogenic survival (Bayala et al., 2014; Franken et al., 2006).

#### 3.2.6 Quantification analysis using the ELISA assay

**FIGURE 12:**
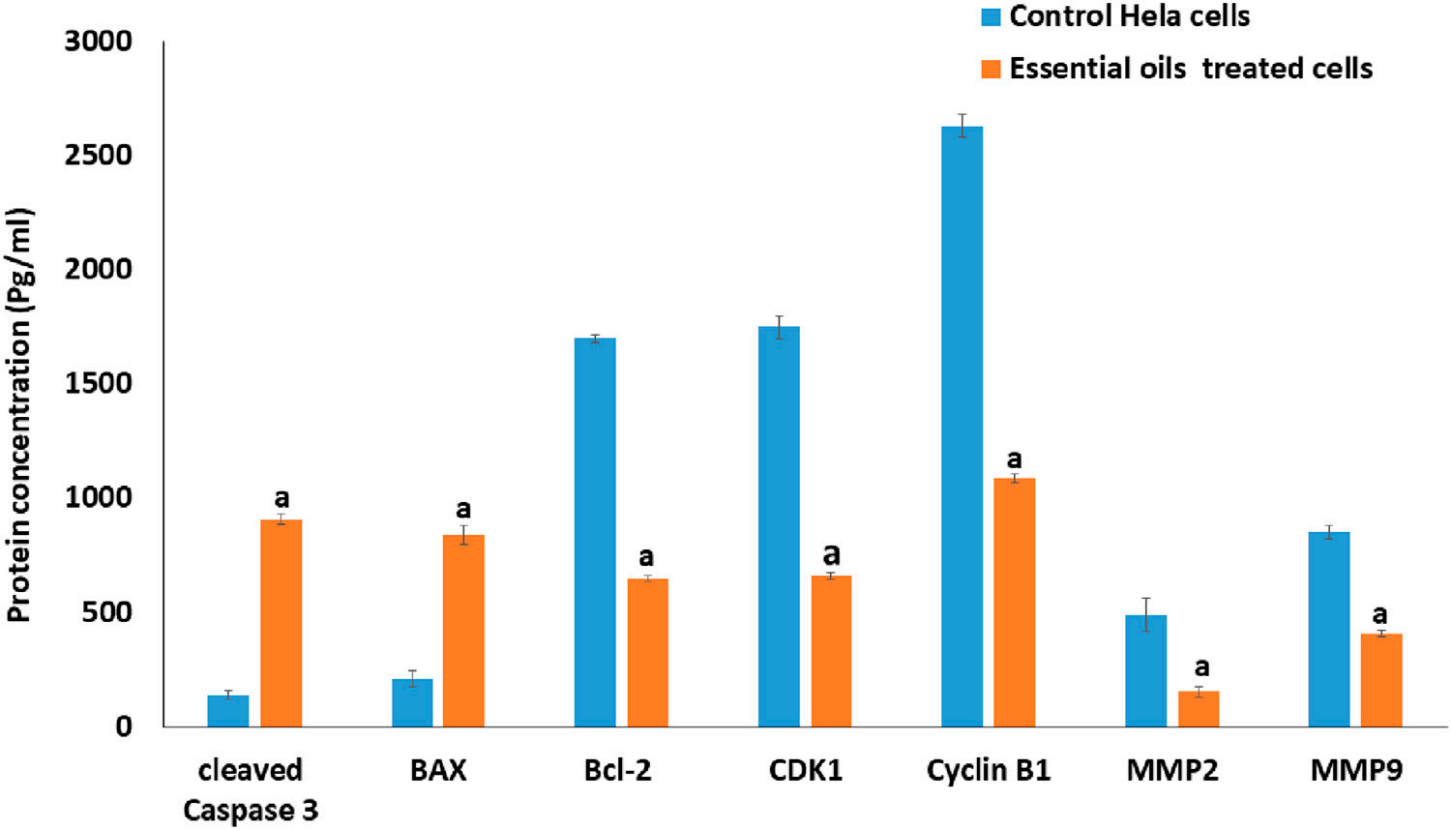
The effect of the IC_50_ dose of *essential oil* on the protein content of cleaved caspase-3, BAX, BCL-2, CDK1, cyclin *B1,* MMP-2, and MMP-9 in HeLa-treated cells compared to the protein content of the control cells, utilizing the ELISA method. The statistical significance was assessed using an independent T-test to compare the studied groups. Statistically significant (*p* < 0.05) differences are shown by lowercase letters. a: (*p* < 0.05) with respect to the control untreated cells.

The expression of metastasis-related proteins, apoptosis-associated proteins, and proteins that control the cell cycle in essential oil-treated cells was quantified and evaluated using the ELISA technique. As seen in Figure 12, the essential oil had a significantly higher influence on the upregulation of protein levels of expression of active cleaved Bax and Caspase 3, coupled with a substantial downregulation of protein levels of Cyclin B1, Bcl-2 CDK1, MMP-9, and MMP-2 in essential oil-treated cells versus untreated ones.

According to the reports (Hanna et al., 2023), the BCL-2 protein family plays a crucial role in regulating apoptosis, with members acting as either antiapoptotic controllers, such as BCL-2, or proapoptotic controllers such as BAX. Where the overexpression of BAX leads to permeabilization of the mitochondria, which leads to enhancing the activation of caspase-3 and stimulates the liberation of cytochrome c to the cytoplasm (Hanna et al., 2023). Consequently, the activation of Caspase-3 leads to DNA damage, apoptosis execution, and a decrease in BCL-2 gene expression levels. In this study, the effect of the prepared essential oil on the expression of apoptosis-related proteins (BCL-2, BAX, and activated Caspase-3) was examined. The findings showed a significant increase in the expression levels of BAX and activated Caspase-3, along with a marked decrease in BCL-2 levels compared to untreated cells (Figure 12). These results suggest that apoptosis induction in the essential oil-treated HeLa cells likely occurred via the mitochondrial pathway.

Also, it is considered that the first important step to prevent the metastasis of cancer cells is the lowering of MMP expression levels (Johansson et al., 2000). To determine whether the observed antimetastatic effect was associated with a reduction in MMP expression, the impact of the essential oil on expression levels of MMP-9 and MMP-2 proteins in HeLa-treated cells was examined. As shown in Figure 12, the essential oil significantly decreased the expression levels of MMP-9 and MMP-2 compared to control cells, suggesting its potential to inhibit HeLa cell migration by reducing these protein levels.

Quantitatively, the migration rate in essential oil–treated cells was reduced to 6.66 µm, compared with 7.89 µm in methotrexate-treated cells. Similarly, wound closure was limited to 38.78% with the essential oil versus 45.9% in methotrexate-treated cells, indicating a stronger inhibition of cell motility. At the molecular level, MMP-2 levels decreased from 490 pg/mL (untreated cells) to 156 pg/mL (≈68% reduction) in treated cells, and MMP-9 levels decreased from 854 pg/mL (untreated cells) to 410 pg/mL (≈52% reduction) in treated cells. These magnitudes of suppression are therapeutically relevant, as even partial inhibition of MMP-2 and MMP-9 has been shown to significantly impair tumor invasion and metastasis (Cathcart et al., 2015; Pavlova et al., 2022).

For context, classical synthetic MMP inhibitors such as marimastat and prinomastat potently inhibit MMP catalytic activity, with reported sub- to low-nanomolar IC_50_ values in biochemical assays (Whittaker et al., 1999). While direct comparison is limited by methodological differences—the data reflect secreted protein concentrations and cellular migration rather than purified enzyme activity—the level of suppression observed with the essential oil falls within ranges reported for other MMP-targeting strategies that attenuate metastatic behavior in preclinical models. Importantly, whereas broad-spectrum MMP inhibitors in clinical trials faced toxicity challenges, the natural product demonstrated a substantial inhibitory effect on MMP expression and migration, suggesting a potentially safer anti-metastatic profile.

Moreover, it has been proposed that inhibition of different Cdk complexes, such as cyclin B/CDK1 and cyclin A/CDK1, at the G2-phase checkpoint creates different regulatory cell cycle checkpoints. Previous studies have shown that DNA damage can inhibit Cdks by preventing Cdk-cyclin complex formation, cyclin downregulation, and limiting tyrosine phosphorylation of Cdks, thus inhibiting their activating phosphorylation. Thus, the inhibition of the CDK1/cyclin B1 complex activity may explain the G2 phase arrest observed in this study. Therefore, the expression levels of cyclin B1 and CDK1 were examined in essential oil-treated cells. The results revealed that the essential oil significantly reduced the expression levels of cyclin B1 and CDK1 (Figure 12), suggesting that the essential oil induced a G2 phase arrest in the treated HeLa cells through inhibition of the activity of the CDK1/cyclin B1 complex.

### 3.3 Antihemolytic effectiveness

The effect of the extracted essential oil on healthy tissues must be confirmed to assess the safety of this extract for therapeutic purposes. The hemolysis test, which accurately depicts how the tested substance interacts with human erythrocytes, is a well-known and frequently used approach for first assessments of the biocompatibility and toxicity of natural substances on normal mammalian cells (1). Subsequently, the biocompatibility of the extracted essential oil against fresh human red blood cells was examined using a hemolysis assay, and the obtained results displayed that the positive control, extracted essential oil, and negative control absorbance values were 0.97 ± 0.05, 0.49 ± 0.03, and 0.43 ± 0.01, respectively. Thus, the hemolysis percentage of the essential oil extracted from C. papaya seeds was 11.1% ± 1.6%, which falls below the generally accepted threshold of 25% for hemocompatible materials (Zhou and Xu, 2015). This indicates that the obtained essential oil demonstrates acceptable safety margins, which confirms the biocompatibility of the extracted essential oil against normal cells.

### 3.4 Molecular docking

**FIGURE 13:**
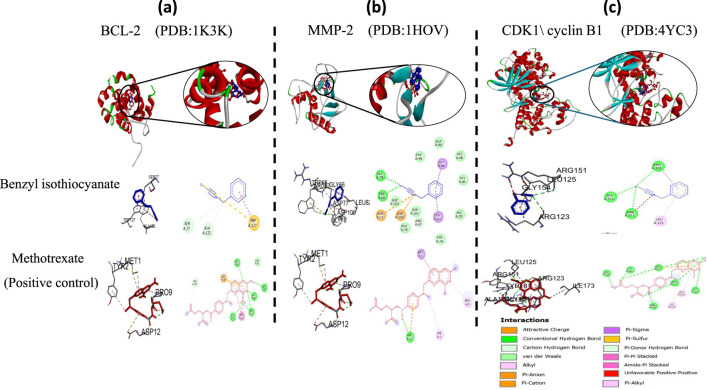
2D and 3D representations of the predicted binding modes of **(a)** BCL2 crystal structure (PDB ID: 1K3K), **(b)** MMP-2 crystal structure (PDB ID: 1HOV), **(c)** CDK1/cyclin B1 crystal structure (PDB ID: 4YC3) for benzyl isothiocyanate (blue), compared to the docked methotrexate (red) as a positive control.

All selected structures met quality standards suitable for reliable docking. For NMR-derived structures, representative conformers were chosen based on structural completeness, low ensemble RMSD, and relevance to the biologically active state, allowing confident prediction of ligand interactions. While for X-ray crystallographic models, resolution values were below 2.5 Å, ensuring accurate atomic positioning. This strategic selection ensures that benzyl isothiocyanate was evaluated against conformations most likely to reflect its physiological binding behavior, thereby reinforcing the validity of the docking results. The docking study of essential oil was carried out with BCL-2, MMP-2, and CDK1/cyclin B1 to determine whether benzyl isothiocyanate would bind to these receptors. It has been known that the BCL-2 protein family has an important role in the apoptosis process (Anilkumar and Bhanu P A, 2022; Alzahrani et al., 2024). Also, it is expected that one of the most important processes to prevent the metastasis of cancer cells is the lowering of MMP protein levels . The docking score, area of contact, and atomic contact energy values of benzyl isothiocyanate compared with standard compounds (methotrexate) were determined. The results showed that benzyl isothiocyanate exhibited higher docking scores with the receptor BCL-2 (−5.52 kcal mol^−^) compared to methotrexate (−5.49 kcal mol^−^); it formed hydrogen bonds with SER77 and ALA122 along with engaging in a pi-pi stacking interaction, as depicted in Figure 13a. While it exhibited the strongest binding score with MMP-2, measuring −6.29 kcal mol^−^ compared to methotrexate (−5.44 kcal mol^−^). Furthermore, it formed a hydrogen bond with GLU78 and TRP68, as shown in Figure 13b. Benzyl isothiocyanate displayed significant inhibition against CDK1/cyclin B1 (−5.75 kcal mol^−^) compared to methotrexate (−5.18 kcal mol^−^). It formed three hydrogen bonds with ARG123, ARG151, and GLY154 in its interaction with CDK1/cyclin B1 (Figure 13c). These interaction studies indicate that the benzyl isothiocyanate present in the essential oil from *C. papaya* seeds could be used as a therapeutic drug against cervical cancer cells. Binding energies obtained from molecular docking were interpreted using established empirical thresholds to assess ligand affinity. Values below −6.0 kcal/mol are generally considered indicative of strong binding potential, while those between −4.0 and −6.0 kcal/mol suggest moderate binding affinity (Hobani et al., 2017). Benzyl isothiocyanate exhibited binding energies in the moderate range, slightly more favorable than methotrexate (which served as a reference inhibitor). Although neither compound reached the strong binding threshold, the comparative docking scores suggest that benzyl isothiocyanate may exert competitive inhibitory effects, reinforce its biological plausibility, and support its potential role as a natural therapeutic candidate.

## 4 Conclusion

Carica papaya is widely consumed for its nutritional and medicinal properties, leading to the generation of considerable seed and peel waste that poses environmental challenges if not effectively utilized. This study demonstrates that essential oil extracted from papaya seeds, rich in benzyl isothiocyanate, exhibits potent anticancer activity against HeLa cervical cancer cells. The oil markedly reduced cell viability (IC_50_ = 16.78 ± 0.38 µg/mL), induced apoptosis as confirmed by Annexin V-FITC/PI analysis, and triggered G_2_/M cell cycle arrest. Moreover, it significantly inhibited cell migration (38.7%), with an efficacy comparable to the standard drug methotrexate (45.9%), and drastically suppressed clonogenic survival, indicating near-complete loss of long-term proliferative capacity. At the molecular level, the oil downregulated proteins involved in apoptosis regulation, cell cycle progression, and metastatic potential. Importantly, it displayed acceptable hemocompatibility, with low hemolytic activity against normal erythrocytes (11.1% ± 1.6%), though comprehensive toxicological evaluations remain necessary.

Collectively, these findings suggest that papaya seed essential oil exerts an apoptosis-dominant cytotoxic effect, supplemented by cytostatic mechanisms including cell cycle arrest and anti-migratory action. This multifaceted activity underscores its strong potential as a natural anti-cervical cancer agent. Nonetheless, further research should focus on elucidating its intracellular signaling targets, pharmacokinetic behavior, and *in vivo* safety profile to support its future therapeutic application.

## Data Availability

The raw data supporting the conclusions of this article will be made available by the authors, without undue reservation.
